# Two New Epoxysteroids from *Helianthus tuberosus*

**DOI:** 10.3390/molecules16108646

**Published:** 2011-10-13

**Authors:** Xiao-Dong Li, Feng-Ping Miao, Nai-Yun Ji

**Affiliations:** Yantai Institute of Coastal Zone Research, Chinese Academy of Sciences, Yantai 264003, China; Email: imnli@163.com (X.-D.L.); fpmiao@yic.ac.cn (F.-P.M.)

**Keywords:** *Helianthus tuberosus*, steroid, 5*α*,8*α*-epidioxy-22*β*,23*β*-epoxyergosta-6-en-3*β*-ol, 5*α*,8*α*-epidioxy-22*α*,23*α*-epoxyergosta-6-en-3*β*-ol

## Abstract

Two new epoxy steroids, 5*α*,8*α*-epidioxy-22*β*,23*β*-epoxyergosta-6-en-3*β*-ol (**1**) and 5*α*,8*α*-epidioxy-22*α*,23*α*-epoxyergosta-6-en-3*β*-ol (**2**), and ten known steroids including (24*R*)-5*α*,8*α*-epidioxyergosta-6-en-3*β*-ol (**3**), (22*E*,24*R*)-5*α*,8*α*-epidioxyergosta-6,22-dien-3*β*-ol (**4**), (22*E*,24*R*)-5*α*,8*α*-epidioxyergosta-6,9(11),22-trien-3*β*-ol (**5**), *β*-sitosterol (**6**), sitost-5-en-3*β*-ol acetate (**7**), 7*α*-hydroxysitosterol (**8**), schleicheol 2 (**9**), (24*R*)-24-ethyl-5*α*-cholestane-3*β*,5*α*,6*β*-triol (**10**), 7*α*-hydroxystigmasterol (**11**), and stigmasterol (**12**) were isolated from *Helianthus tuberosus* grown in Laizhou salinized land of coastal zone of Bohai Sea, China. The structures of these compounds were unambiguously established by 1D, 2D NMR and mass spectroscopic techniques. The new compounds **1** and **2** exhibited weak antibacterial activity and no antifungal activity.

## 1. Introduction

**Figure 1:**
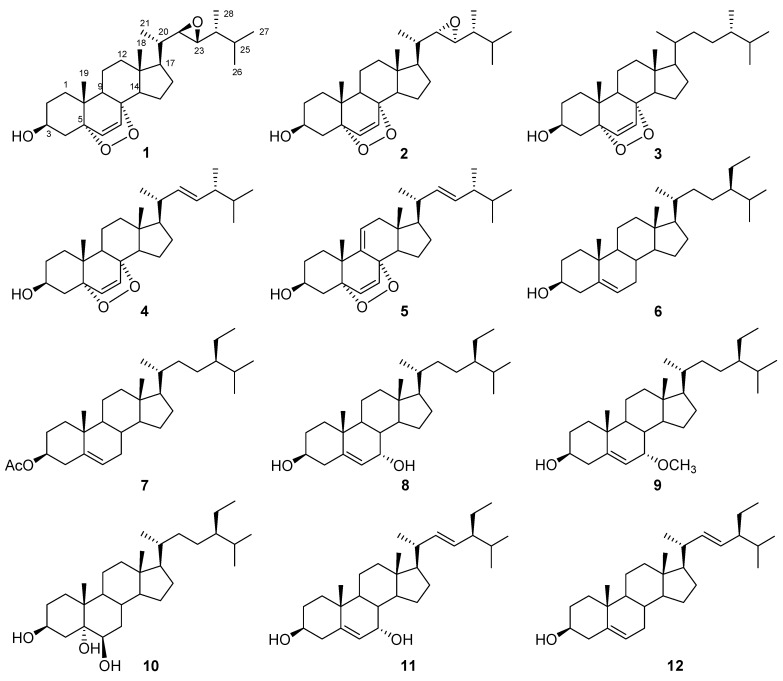
Steroids **1–12** from *H. tuberosus.*

*Helianthus tuberosus* Linn (Asteraceae, commonly named Jerusalem artichoke) is an herbaceous plant cultivated widely around the temperature areas for its edible tubers. In addition, it is widely used in industry as a raw material to produce inulin and ethanol [1,2]. Phytochemical investigations have indicated that this species is a rich source of sesquiterpenes and diterpenes [3,4]. Triterpenes and steroids have also been reported from this species [5,6]. Recently, *H. tuberosus* has been successfully planted in Laizhou salinized land for ameliorating the salizined soil, where the salt contents and pH values are 3.79 g/kg and 7.55 at 0–20 cm depth and 4.01 g/kg and 7.50 at 20–40 cm depth, respectively [7]. In order to explore the application of this grown plant, its secondary metabolites were examined. As a result, two new epoxysteroids, 5*α*,8*α*-epidioxy-22*β*,23*β*-epoxyergosta-6-en-3*β*-ol (**1**) and 5*α*,8*α*-epidioxy-22*α*,23*α*-epoxyergosta-6-en-3*β*-ol (**2**), and ten known steroids including (24*R*)-5*α*,8*α*-epidioxyergosta-6-en-3*β*-ol (**3**) [8], (22*E*,24*R*)-5*α*,8*α*-epidioxyergosta-6,22-dien-3*β*-ol (**4**) [9], (22*E*,24*R*)-5*α*,8*α*-epidioxyergosta-6,9(11),22-trien-3*β*-ol (**5**) [9], *β*-sitosterol (**6**) [10,11], sitost-5-en-3*β*-ol acetate (**7**) [11], 7*α*-hydroxysitosterol (**8**) [11], schleicheol 2 (**9**) [12], (24*R*)-24-ethyl-5*α*-cholestane-3*β*,5*α*,6*β*-triol (**10**) [13], 7*α*-hydroxystigmasterol (**11**) [14], stigmasterol (**12**) [10] were isolated and identified (Figure 1). Herein we mainly report the isolation, structure elucidation, and bioactivity of steroids **1**–**12**.

## 2. Results and Discussion

**Figure 2:**
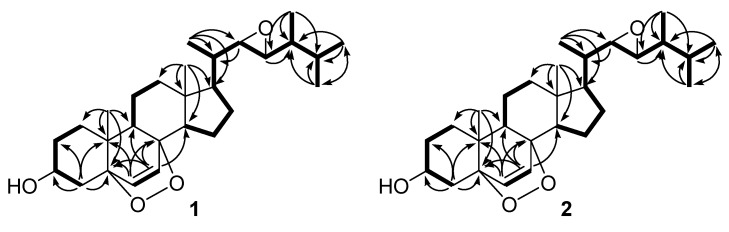
Key HMBC (curved arrows) and ^1^H–^1^H COSY (bold lines) correlations of **1** and **2**.

Compound **1** was obtained as a white solid. The broad IR absorption at *v*_max_ 3,410 cm^−1^ suggested the presence of a hydroxyl group in the molecule. The molecular formula was determined to be C_28_H_44_O_4_ on the basis of HREIMS (*m/z* 444.3238 [M]^+^, calcd. for C_28_H_44_O_4_, 444.3240), indicating seven degrees of unsaturation. The ^1^H-NMR spectrum (Table 1) showed two methyl singlets, four methyl doublets, two double doublets assigned to two epoxygenated methines, one multiplet characteristic of an oxygenated methine, and two doublets attributed to two olefinic protons. The ^13^C- NMR and Distortionless Enhancement by Polarization Transfer (DEPT) spectra (Table 1) along with the HSQC experiment displayed the presence of six methyls, seven methylenes, eleven methines including one oxygenated methine (C-3), two epoxygenated methines (C-22 and C-23), and two *sp*^2^ methines (C-6 and C-7), and four quaternary carbon atoms containing two oxygenated carbons (C-5 and C-8). Detailed NMR data comparison with those reported for (24*R*)-5*α*,8*α*-epidioxyergosta-6-en-3*β*-ol (**3**) revealed that **1** differed from **3** mainly at the side chain moiety [8]. The 5*α*,8*α*-epidioxy moiety was further confirmed by the ^1^H- and ^13^C-NMR data comparison with those of ergosta-6,22-dien-3,5,8-triol and 5*β*,8*β*-epidioxyergosta-6-en-3*β*-ol [9,15]. Replacing two methylenes at C-22 and C-23 in **3**, two epoxy methines were located at C-22 and C-23 in **1** by the HMBC correlations from H-21 to C-17, C-20, and C-22, from H-22 to C-20, from H-23 to C-24, and from H-28 to C-23, C-24, and C-25 and ^1^H–^1^H COSY correlations between H-20/H-22, H-22/H-23, and H-23/H-24. The relative configuration for the side chain moiety of **1** were established by the identical NMR data with those reported for (22*R*,23*R*,24*R*)-24-methyl-22,23-epoxy-3*α*,5-cyclo-5*α*-cholestan-6*β*-yl acetate [16]. Furthermore, the NOESY correlation between H-20/H-23 indicated C-20 and H-23 to be on the same side of the epoxy ring. The NOESY correlations of H-22 with H-17 and H-21 allowed them to be the same orientation, while H-23 and C-28 were assigned on the same face by the observed NOESY correlation between H-23/H-28. So, compound **1** was identified as 5*α*,8*α*-epidioxy-22*β*,23*β*-epoxy-ergosta-6-en-3*β*-ol, which was verified by the other HMBC, ^1^H–^1^H COSY (Figure 2), and NOESY correlations.

Compound **2** was also obtained as a white solid. The broad IR absorption at *v*_max_ 3,402 cm^−1^ indicated the presence of a hydroxyl group in the molecule. The molecular formula was also established to be C_28_H_44_O_4_ on the basis of HREIMS (*m/z* 444.3238 [M]^+^, calcd. for C_28_H_44_O_4_, 444.3240), implying seven degrees of unsaturation. The ^1^H-NMR spectrum (Table 1) exhibited two methyl singlets, four methyl doublets, two double doublets representative of two epoxygenated methines, one multiplet ascribed to an oxygenated methine, and two doublets assignable to two olefinic protons. The ^13^C-NMR and DEPT spectra (Table 1) along with the HSQC experiment revealed the presence of six methyls, seven methylenes, eleven methines, and four quaternary carbon atoms. The NMR data showed close similarity to those of **1**, with the exception of the different resonances for side chain moiety. The identical NMR data of the side chain moiety for **2** with those reported for (22*S*,23*S*,24*R*)-24-methyl-22,23-epoxy-3*α*,5-cyclo-5*α*-cholestan-6*β*-yl acetate and the observed HMBC and ^1^H–^1^H COSY correlations (Figure 2) confirmed **2** to be 5*α*,8*α*-epidioxy-22*α*,23*α*-epoxyergosta-6-en-3*β*-ol, an isomer of **1** [16]. The observed NOESY correlations further confirmed the relative configuration for the side chain moiety. In the NOESY spectrum, the correlation between H-20/H-23 located C-20 and H-23 on the same face of the epoxy ring. The correlations of H-22 with H-17 and H-21 positioned them in the same direction, while H-23 and C-28 were placed on the same side by the correlation between H-23/H-28.

The antimicrobial activity of new epoxy steroids **1** and **2** were evaluated using a standard agar diffusion test at 30 μg/disk. Compound **1** showed weak inhibitory activity against *Escherichia coli* and *Staphylococcus aureus* (inhibition diameter 7 mm), and **2** exhibited weak inhibitory activity against *E. coli* (inhibition diameter 7 mm). However, **1** and **2** were found no antifungal activity against plant pathogens *Colletotrichum lagenarium* and *Fusarium oxysporium*. Additionally, **1** and **2** exhibited inhibitory rates of 58.7% and 22.5%, respectively, in the toxicity assay against brine shrimp (*Artemia salina*) at 100 μg/mL.

**Table 1:** ^1^H and ^13^CNMR data for **1** and **2** (in CDCl_3_, *δ* in ppm, *J* in Hz).

No.	**1**			**2**	
	*δ*_H_	*δ*_C_		*δ*_H_	*δ*_C_
1a	1.70 (m)	34.7 (CH_2_)		1.69 (m)	34.7 (CH_2_)
1b	1.95 (m)			1.95 (m)	
2a	1.54 (m)	30.1 (CH_2_)		1.55 (m)	30.1 (CH_2_)
2b	1.85 (m)			1.84 (m)	
3	3.97 (m)	66.4 (CH)		3.97 (m)	66.4 (CH)
4a	1.91 (m)	36.9 (CH_2_)		1.91 (m)	36.9 (CH_2_)
4b	2.12 (ddd, 13.8, 4.9, 1.4)			2.12 (ddd, 13.8, 5.0, 1.8)	
5		82.2 (C)			82.2 (C)
6	6.26 (d, 8.5)	135.6 (CH)		6.25 (d, 8.5)	135.5 (CH)
7	6.49 (d, 8.5)	130.5 (CH)		6.50 (d, 8.5)	130.6 (CH)
8		79.3 (C)			79.4 (C)
9	1.51 (m)	51.1 (CH)		1.51 (m)	51.2 (CH)
10		37.0 (C)			37.0 (C)
11a	1.23 (m)	23.4 (CH_2_)		1.24 (m)	23.4 (CH_2_)
11b	1.53 (m)			1.52 (m)	
12a	1.28 (m)	39.3 (CH_2_)		1.25 (m)	39.4 (CH_2_)
12b	1.98 (m)			1.93 (m)	
13		45.0 (C)			45.0 (C)
14	1.57 (m)	51.3 (CH)		1.57 (m)	51.3 (CH)
15a	1.47 (m)	21.0 (CH_2_)		1.48 (m)	20.8 (CH_2_)
15b	1.68 (m)			1.68 (m)	
16a	1.43 (m)	28.0 (CH_2_)		1.71 (m)	27.1 (CH_2_)
16b	1.96 (m)			2.01 (m)	
17	1.37 (m)	53.9 (CH)		1.40 (m)	56.3 (CH)
18	0.79 (s)	12.7 (CH_3_)		0.79 (s)	12.8 (CH_3_)
19	0.89 (s)	18.2 (CH_3_)		0.88 (s)	18.2 (CH_3_)
20	1.17 (m)	39.2 (CH)		1.31 (m)	38.2 (CH)
21	1.08 (d, 6.4)	16.9 (CH_3_)		0.99 (d, 7.0)	16.0 (CH_3_)
22	2.38 (dd, 8.2, 1.9)	62.8 (CH)		2.58 (dd, 7.1, 2.2)	63.8 (CH)
23	2.66 (dd, 8.4, 1.9)	63.9 (CH)		2.45 (dd, 7.8, 2.2)	60.3 (CH)
24	1.09 (m)	42.4 (CH)		1.05 (m)	42.2 (CH)
25	1.78 (m)	31.0 (CH)		1.65 (m)	31.1 (CH)
26	0.92 (d, 6.8)	18.6 (CH_3_)		0.92 (d, 6.8)	19.5 (CH_3_)
27	0.96 (d, 6.8)	20.2 (CH_3_)		0.95 (d, 6.8)	20.4 (CH_3_)
28	0.91 (d, 7.0)	12.6 (CH_3_)		0.97 (d, 6.9)	13.6 (CH_3_)

## 3. Experimental

### 3.1. General

NMR spectra were recorded at 500 and 125 MHz for ^1^H and ^13^C, respectively, on a Bruker Avance III 500 NMR spectrometer in CDCl_3_ using TMS as internal standard. Low and high resolution mass spectra were determined on an Autospec Premier P776 mass spectrometer. IR spectra were obtained on a JASCO FT/IR-4100 Fourier Transform InfraRed spectrometer. HPLC separation was carried out on an Elite HPLC system (P270 pump, UV230+ detector, Dalian Elite Analytical Instruments Co., Ltd, Dalian, China) using an Eclipse XDB-C18 (5μm, 9.4 × 250 mm) column. Column chromatography was performed with silica gel (100–200 and 200–300 mesh, Qingdao Haiyang Chemical Co., Qingdao, China) and Sephadex LH-20 (Pharmacia). Precoated silica gel plates (GF-254, Qingdao Haiyang Chemical Co., Qingdao, China) were used for preparative TLC purification. All solvents were of analytical grade.

### 3.2. Plant Material

*Helianthus tuberosus* Linn was grown by Qin-Tai Zhao in Laizhou salinized land of coastal zone of Bohai Sea, China, which was collected in December, 2008. A voucher specimen (SP0812) has been deposited at the Bio-Resource Laboratory of Yantai Institute of Coastal Zone Research, Chinese Academy of Sciences.

### 3.3. Extraction and Isolation

Extraction and isolation of the leaves: the dried and powdered sample (1.1 kg) was extracted exhaustively with 95% aqueous EtOH (5 L, 24 h, 25 °C). The concentrated extract was partitioned between H_2_O and EtOAc. The EtOAc-soluble fraction (19.1 g) was subjected to silica gel column chromatography [CC, gradient of EtOAc in petroleum ether (PE) (0–100%)] to give nine fractions (Frs. I–IX), monitored by TLC. Fr. III eluted with PE/EtOAc (20:1) and was further purified by CC on silica gel (PE/EtOAc, 20:1) and Sephadex LH-20 (CHCl_3_/MeOH, 1:1) and preparative TLC (PE/CHCl_3_, 3:1) to yield **4** (16.6 mg) and **5** (3.1 mg). Fr. V eluted with PE/EtOAc (10:1) too and was further purified by CC on silica gel (PE/EtOAc, 9:1) and Sephadex LH-20 (CHCl_3_/MeOH, 1:1) to afford **6** (13.5 mg), **12** (24.6 mg). Fr. VIII eluted with EtOAc and was further purified by CC on silica gel (PE/EtOAc, 1:1) and Sephadex LH-20 (CHCl_3_/MeOH, 1:1) to give **8** (4.5 mg) and a subfraction, which was further purified by preparative HPLC (MeOH/H_2_O, 4:1) to yield **11** (3.7 mg). 

Extraction and isolation of the tubers: the dried and powdered sample (16.0 kg) was extracted with 95% aqueous EtOH (50 L, 3 d, 25 °C), then partitioned between H_2_O and EtOAc. The EtOAc-soluble fraction (53.0 g) was chromatographed over silica gel column using stepwise gradient of PE/EtOAc to yield twenty-six fractions (Frs. 1–26), based on TLC analysis. Fr. 6 eluted with PE/EtOAc (50:1) and was further purified by CC on Sephadex LH-20 (CHCl_3_/MeOH, 1:1) and preparative TLC (PE/EtOAc, 40:1) to give **7** (11.2 mg). Fr. 12 eluted with PE/EtOAc (5:1) and was further purified by CC on silica gel (PE/EtOAc, 5:1) and preparative HPLC (MeOH/H_2_O, 17:3) to afford **6** (1.8 mg), **8** (2.7 mg), **9** (1.5 mg), **12** (7.5 mg), and a subfraction, which was further purified by preparative TLC (CHCl_3_/EtOAc, 3:1) to give **10** (10.2 mg). Fr. 17 eluted with PE/EtOAc (2:1) and was purified by CC on silica gel (PE/EtOAc, 3:1) and Sephadex LH-20 (CHCl_3_/MeOH, 1:1) and preparative HPLC (MeOH/H_2_O, 3:1) to yield **1** (3.2 mg, t_R_ 37 min), **2** (3.3 mg, t_R_ 41 min), and **3** (2.7 mg, t_R_ 55 min).

*5α,8α-Epidioxy-22β,23β-epoxyergosta-6-en-3β-ol* (**1**): White solid; [α]^16^_D_ –18.7 (c 0.088, MeOH); IR (KBr) v_max_ 3410, 2954, 2881, 1600, 1462, 1381, 1041 cm^−1^; ^1^H- and ^13^C-NMR data, see Table 1; EIMS m/z (%) 444 (8), 426 (9), 412 (100), 379 (17), 152 (50); HREIMS: m/z 444.3238 [M]^+^ (calcd. for C_28_H_44_O_4_, 444.3240).

*5α,8α-Epidioxy-22α,23α-epoxyergosta-6-en-3β-ol* (**2**): White solid; [α]^16^_D_ –58.7 (c 0.050, MeOH); IR (KBr) v_max_ 3402, 2954, 2881, 1624, 1458, 1381, 1030 cm^−1^; ^1^H- and ^13^C-NMR data, see Table 1; EIMS m/z (%) 444 (20), 426 (22), 412 (100), 379 (33), 268 (36), 152 (51); HREIMS: m/z 444.3238 [M]^+^ (calcd. for C_28_H_44_O_4_, 444.3240).

### 3.4. Bioassays

Antibacterial and antifungal activities were assayed using chloramphenicol as positive control with inhibition diameters of 28 and 30 mm for *E. coli* and *S. aureus*, respectively, as described previously [17]. Toxicity against brine shrimp (*Artemia salina*) was also tested as described previously [18].

## 4. Conclusions

Enriched steroids with ergosterol (**1**–**5**), sitosterol (**6**–**10**), and stigmasterol (**11**, **12**) skeletons were isolated and identified from *H. tuberosus* planted in coastal salinized land, and ergosterol derivatives were reported from this species for the first time. The isolation of two new epoxy sterols (**1**, **2**) was a new addition to the molecular diversity of *H. tuberosus*, which exhibited weak antibacterial activity and toxicity against brine shrimp.
